# Kinetic Theory with Casimir Invariants—Toward Understanding of Self-Organization by Topological Constraints

**DOI:** 10.3390/e27010005

**Published:** 2024-12-25

**Authors:** Zensho Yoshida

**Affiliations:** National Institute for Fusion Science, Oroshi, Toki 509-5292, Gifu, Japan; yoshidazensho@gmail.com

**Keywords:** self-organization, topological constraint, Casimir invariant, noncanonical Hamiltonian system, co-adjoint representation

## Abstract

A topological constraint, characterized by the Casimir invariant, imparts non-trivial structures in a complex system. We construct a kinetic theory in a constrained phase space (infinite-dimensional function space of macroscopic fields), and characterize a self-organized structure as a thermal equilibrium on a leaf of foliated phase space. By introducing a model of a grand canonical ensemble, the Casimir invariant is interpreted as the number of topological particles.

## 1. Introduction

Every physics theory is based on two fundamental principles, i.e., the action principle and the entropy principle. Each principle dictates the extremal at two different poles; one rules how an individual element moves, while the other determines the most probable state in a system of many elements. Understanding how these two principles describe actual complex systems is still a challenging problem; although the statistical mechanics of gas particles has been successful in describing thermodynamical quasi-static processes, more spectacular macroscopic phenomena are often observed in fluids, especially in plasmas, which seem at first glance to contradict the expectation of thermal death. This article is an attempt to construct a model by which we may explain the emergence of a collective structure in a complex system—the self-organization of macroscopic structures in a plasma is an example to test the model.

It is widely recognized that plasmas are more capable of creating structures than ordinary gases. Such structures are epitomized by *vortices*, which are dynamical structures pertinent to momentum rather than energy, and hence, they are fundamentally different from *clusters* that are created by some gravitational energy. Interestingly, a vortex works as a spontaneous field that imparts a constraint on the dynamics of the system—the constraint is represented by some conservation law that is independent of the energy conservation law. The emergence of non-trivial structures, resisting against the entropy production that leads to thermal death, is thought to be enabled by constraints on the possible dynamics.

The *magnetic helicity* plays such a role. The Taylor relaxed state is a simple model for such a self-organized structure in a magnetized plasma, which minimizes the energy of magnetofluid for a given (fixed) magnetic helicity [1,2]. Behind is a hypothesis that the thermal energy of particles bears the entropy of the total system, being complementary with respect to the macroscopic phase space of magnetic and fluid velocity. However, such separability is unclear since the complexity in the macroscopic phase space, appearing as turbulence, may also be subject to some entropy principle. Here, we construct a statistical mechanical theory, and explain a self-organized structure as a thermodynamic equilibrium in a phase space of macroscopic fields, which subsumes the entropy of microscopic particles as one of the macroscopic fields.

The key to formulating an appropriate phase space is Liouville’s theorem that guarantees the invariance of statistical measure through every transition in the phase space. The ideal fluid/plasma models are known to be *noncanonical* Hamiltonian systems [3,4,5,6,7,8,9,10], for which the relevant invariant measures are not simple because they are dependent on the field variables, reflecting the noncanonicality. However, we may re-formulate them as *reductions* of canonical Hamiltonian systems in some phase spaces of potential variables [11,12,13], i.e., we may regard a fluid/plasma system as a subsystem of an inflated canonical system. For a canonical Hamiltonian system, the invariant measure is just the $L^{2}$ measure of the canonical variables that span the symplectic manifold. *Casimir invariants* guide us to relate the original noncanonical system and its canonicalized larger system; a Casimir invariant generates a gauge group unchanging original observables.

This paper is organized as follows: Section 2 is devoted for a concise review of Hamiltonian mechanics. Having a Casimir invariant is the definition of noncanonicality [7,8]. Invoking an example of plasma confinement in a magnetosphere (Section 2.2), we elucidate a relation between a Casimir invariant and an adiabatic invariant, and show how a reduction in the phase space is related to a *topological constraint* imposed by the conservation of a Casimir invariant, or an adiabatic invariant [14,15]. In Section 3, we formulate a macroscopic (fluid–mechanical) model of plasmas as an infinite-dimensional (field-theoretic) Hamiltonian system, which is noncanonical, and is endowed with the magnetic helicity as its Casimir invariant. In Section 3.2, we recast it into a canonicalized system by *Clebsch parameterizing* the physical variables [13,16,17,18,19,20,21,22,23]. The canonicalized formulation provides us with an appropriate phase space of Clebsch parameters, in which we can argue the entropy principle with a rigorous invariant measure. In Section 4, we formulate a kinetic theory of macroscopic fields, and characterize the Taylor relaxed state as a thermal equilibrium maximizing the entropy in the phase space of canonical variables. By introducing a model of grand canonical ensemble, the Casimir invariant, i.e., helicity, is interpreted as the number of *topological particles* that carry the local helicity as the topological charge [24].

## 2. Hamiltonian Structure and Foliated Phase Space

This section is a preparation of the general theory of phase space foliation, with an example of finite-dimensional Hamiltonian mechanics which demonstrates how a topological constraint emerges from coarse-graining.

### 2.1. General Theory

We start by reviewing the basic formalism of Hamiltonian mechanics and the Poisson manifold. Let *Z* be the phase space in which a state vector *u* resides. Observables are members of the space $g=C^{\infty }\left(Z\right)$ of smooth real-valued functionals on *Z*. For F(u), we define its gradient $\partial_{u}F$ ($\in Z^{*}$) by

(1) $$F\left(u+\epsilon \tilde{u}\right)-F\left(u\right)=\epsilon \langle \partial_{u}F,\tilde{u}\rangle +O\left(\epsilon^{2}\right)\;\left(\forall \tilde{u}\in Z\right),$$

where 〈,〉 is the pairing $Z^{*}\times Z\to R$. A bilinear map g×g→g such that

(2) $$\left\{F,G\right\}=\langle \partial_{u}F,J\partial_{u}G\rangle$$

is called a Poisson bracket (defined by a Poisson operator $J:Z^{*}\to Z$), if it is antisymmetric and satisfies Jacobi’s identity (i.e., g is a Lie algebra endowed with the bracket {,}). We may allow the Poisson operator *J* to be a function of *u*, and then we write J(u).

The dynamics in g is dictated by

(3) $$\dot{F}=\left\{F,H\right\},$$

where $\dot{F}$ means the rate of change of *F* induced by a *Hamiltonian H*. Regarding {∘,H} as a map g→g, we write ${\mathrm{ad}}_{H}\circ =\left\{\circ ,H\right\}$, which gives the *adjoint representation* of the Lie algebra g.

By $\dot{F}=\langle \partial_{u}F,\dot{u}\rangle$, we observe that (3) is equivalent to

(4) $$\dot{u}=J\left(u\right)\partial_{u}H\left(u\right),$$

which is the general form of Hamilton’s equation of motion.

A prototypical Poisson bracket is endowed with the *symplectic* Poisson operator such that

(5) $$J_{c}=\left(\begin{array}{cc} 0 & I\\ -I & 0 \end{array}\right).$$

Then, Equation (4) is the textbook Hamilton’s equation.

While $J_{c}$ is regular (full rank), a more general Poisson operator may have a non-trivial kernel. If an element v∈Ker(J) is integrable to yield a functional C(u) such that $v=\partial_{u}C\left(u\right)$, we observe that {C,G}=0 for all *G*, implying that such an observable C(u) cannot be changed by any Hamiltonian. Such a functional C(u) is called a *Casimir invariant*; the level sets of C(u), which we call Casimir leaves, foliate the phase space *Z*. A Hamiltonian system that has a non-trivial Ker(J) is said to be *noncanonical*.

**Remark** **1** (Canonicalization around Casimir invariants). *By a simple example of a noncanonical Poisson bracket, we show how a Casimir invariant foliates the phase space, and how we can canonicalize such a Poisson bracket. Let $u_{3}={\left(x,y,z\right)}^{T}\in Z_{3}=R^{3}$, and, for $F(u_{3})$ and $G(u_{3})$,*

(6) $${\left\{F,G\right\}}_{3}=\langle \partial_{u_{3}}F,J_{3}\partial_{u_{3}}G\rangle ,\;\mathrm{with}\;J_{3}=\left(\begin{array}{ccc} 0 & 0 & 0\\ 0 & 0 & 1\\ 0 & -1 & 0 \end{array}\right).$$

*We easily find that ${\left\{\;,\;\right\}}_{3}$ is a noncanonical Poisson bracket, and C=f(x) (f is an arbitrary smooth function) is a Casimir invariant, i.e., ${\left\{f\left(x\right),G\right\}}_{3}=f^{\prime }\left\{x,G\right\}=0$ for all G. Therefore, every orbit (for any Hamiltonian) is constrained on a surface x=c (the constant number c is determined by the initial condition). Such a surface (leaf) $Z_{2}=\left\{u_{2}={\left(y,z\right)}^{T};\;y,z\in R\right\}$, i.e., a level set of the Casimir invariant C=f(x), is the effective phase space. On $Z_{2}$, the noncanonical bracket ${\left\{\;,\;\right\}}_{3}$ reads as*

(7) $${\left\{F,G\right\}}_{2}=\langle \partial_{u_{2}}F,J_{2}\partial_{u_{2}}G\rangle ,\;\mathrm{with}\;J_{2}=\left(\begin{array}{cc} 0 & 1\\ -1 & 0 \end{array}\right).$$

*Therefore, the leaf $Z_{2}$ is a symplectic manifold. Here, we canonicalize $J_{3}$ by separating its kernel. There is the other method of canonicalization; instead of separating the kernel, we expand the dimension. We embed $Z_{3}$ in $Z_{4}=\left\{u_{4}={\left(\theta ,u_{3}\right)}^{T};\;\theta \in R,\;u_{3}\in Z_{3}\right\}$, and define, for $F(u_{4})$ and $G(u_{4})$,*

(8) $${\left\{F,G\right\}}_{4}=\langle \partial_{u_{4}}F,J_{4}\partial_{u_{4}}G\rangle ,\;\mathrm{with}\;J_{4}=\left(\begin{array}{cccc} 0 & 1 & 0 & 0\\ -1 & 0 & 0 & 0\\ 0 & 0 & 0 & 1\\ 0 & 0 & -1 & 0 \end{array}\right).$$

*In this 4-dimensional canonical system, we observe ${\left\{F,G\right\}}_{4}={\left\{F,G\right\}}_{3}$ if F and G are independent of the added variable θ. This restricted situation is said to be the reduction. In the present example, the nullity of $J_{3}$ is obvious, so both methods of canonicalization apply easily. While the kernel of a general noncanonical Poisson operator J may not be apparent, Darboux’ theorem guarantees that we can always transform J into the standard form such that $0⊕J_{c}⊕\cdots ⊕J_{c}$ (at least locally in the phase space). At the kernel are the Casimir invariants, so finding Casimir invariants is the key to transform J into the standard form. In Section 3.2, we see a rather sophisticated example of reduction in canonicalizing a plasma model.*

### 2.2. Example: Casimir Invariant and Self-Organization on a Foliated Phase Space

#### 2.2.1. Magnetic Confinement

There are two different types of structures in the universe, which are created by different mechanisms. Gravity confines particles and creates stars. Magnetic fields also confine plasmas and create stellar magnetospheres. However, the mechanism of magnetic confinement is not so simple. The magnetic force does not change the energy, and hence, the Boltzmann distribution, for instance, is independent of the magnetic field. Then, how can the magnetic confinement be possible? While the gravity (and electrostatic force) is represented by a potential energy, the magnetic force is represented by a *potential momentum* appearing in the canonical momentum of Hamiltonian mechanics. Here, we show how it achieves *confinement* [14,15,25].

Let us consider a dipole magnetic field as a simple and naturally made confinement system (imagine a stellar magnetosphere). A dipole magnetic field can be written as B=∇ψ×∇θ (ψ is the flux function, and θ is the toroidal angle; $\psi =rA_{\theta }$, where $A_{\theta }$ is the θ component of the vector potential). In a strong axisymmetric magnetic field, the canonical angular momentum $P_{\theta }$ is dominated by the magnetic part qψ (*q* is the charge). Therefore, the conservation of $P_{\theta }\approx q\psi$ restricts a particle to move on a magnetic surface (a level set of ψ). If fluctuations of the electromagnetic field violate the axisymmetry $\partial_{\theta }=0$, however, particles can diffuse across magnetic surfaces. Interestingly, in a dipole magnetic field of magnetosphere, the diffusion proceeds to steepen the density gradient, seemingly contradicting the entropy principle. Here, we show that such a phenomenon is consistent with the entropy principle if we evaluate the appropriate entropy that is consistent to the Hamiltonian mechanics of magnetized particles.

#### 2.2.2. Topological Constraints Imposed by Adiabatic Invariants

The Hamiltonian of a charged particle is

(9) $$H=\frac{m}{2}v^{2}+q\phi ,$$

where v=(P−qA)/m is the velocity, P is the canonical momentum, (ϕ,A) is the electromagnetic 4-potential, *m* is the particle mass, and *q* is the charge. In the later discussion, we will neglect ϕ, assuming charge neutrality. Denoting by $v_{\|}$ and $v_{⊥}$ the parallel and perpendicular (with respect to the local magnetic field) components of the velocity, we may write

(10) $$H=\frac{m}{2}v_{⊥}^{2}+\frac{m}{2}v_{\|}^{2}+q\phi .$$

The velocities are related to the mechanical momentum as p=mv, $p_{\|}=mv_{\|}$, and $p_{⊥}=mv_{⊥}$.

In a strong magnetic field, $v_{⊥}$ can be decomposed into a small-scale cyclotron motion $v_{c}$ and a macroscopic *guiding-center* drift motion $v_{d}$. The periodic cyclotron motion $v_{c}$ can be written as $\left(m/2\right)v_{c}^{2}=\mu \omega_{c}\left(x\right)$ in terms of the magnetic moment μ and the cyclotron frequency $\omega_{c}\left(x\right)$; the adiabatic invariant μ and the gyration phase $\vartheta_{c}=\omega_{c}t$ constitute an action–angle pair.

Let ζ be a longitudinal coordinate along magnetic field lines. We define a *magnetic coordinate system* (ψ,ζ,θ). The kinetic energy of drift motion is $mv_{d}^{2}/2={\left(P_{\theta }-q\psi \right)}^{2}/\left(2mr^{2}\right)$, where $P_{\theta }$ is the canonical angular momentum in the θ direction and *r* is the radius from the geometric axis. In terms of the canonical variables $z={\left(\vartheta_{c},\mu ,\zeta ,p_{\|},\theta ,P_{\theta }\right)}^{T}$, the Hamiltonian of a magnetized particle reads

(11) $$H_{\mathrm{gc}}=\mu \omega_{c}+\frac{1}{2m}p_{\|}^{2}+\frac{1}{2m}\frac{{\left(P_{\theta }-q\psi \right)}^{2}}{r^{2}}+q\phi .$$

Here, the energy of the cyclotron motion is written as $\mu \omega_{c}\left(x\right)$ by coarse-graining the gyro-phase $\vartheta_{c}$.

Now we formulate the *macro hierarchy* on which the thermal equilibrium creates a non-trivial structure [14,15]. The adiabatic invariance of the magnetic moment μ imposes a topological constraint on the motion of particles. We observe that such a topological constraint foliates the phase space by the level set of μ—to see the relation between the adiabatic invariant and Casimir leaves, we start with the general (micro–macro total) phase space, and then separate the microscopic action–angle pair μ-$\vartheta_{c}$; the *macro phase space* is the remaining submanifold (leaf) immersed in the general phase space, on which the adiabatic invariant μ is viewed as a Casimir invariant.

The Poisson bracket on the total phase space, spanned by the canonical variables $z={\left(\vartheta_{c},\mu ,\zeta ,p_{\|},\theta ,P_{\theta }\right)}^{T}$, is $\left\{F,G\right\}=\langle \partial_{z}F,J\partial_{z}G\rangle$, where J is the canonical 6×6 symplectic matrix (with $J_{c}$ of (5)):

(12) $$J=J_{c}⊕J_{c}⊕J_{c}.$$

Liouville’s theorem for the canonical Hamiltonian system guarantees the invariance of the natural measure $d^{6}z$, by which we obtain the standard Boltzmann distribution.

To extract the macro hierarchy, we separate the microscopic variable $\vartheta_{c}$ and reduce the state vector z to $z_{\mathrm{gc}}={\left(\mu ,\zeta ,p_{\|},\theta ,P_{\theta }\right)}^{T}$ which describes the dynamics of a guiding center. The gradient $\partial_{z_{\mathrm{gc}}}F$ is now a 5-dimensional vector. We define a degenerate 5×5 Poisson matrix

(13) $$J_{\mathrm{gc}}=0⊕J_{c}⊕J_{c}.$$

The modified Poisson bracket ${\left\{F,G\right\}}_{\mathrm{gc}}={\langle \partial_{z_{\mathrm{gc}}}F,J_{\mathrm{gc}}\partial_{z_{\mathrm{gc}}}G\rangle }_{\mathrm{gc}}$ determines the kinematics on the macro hierarchy; the corresponding kinetic equation $\dot{f}+{\left\{H_{\mathrm{gc}},f\right\}}_{\mathrm{gc}}=0$ reproduces the familiar drift-kinetic equation (for example [26]).

The nullity of $J_{\mathrm{gc}}$ makes the Poisson bracket ${\left\{\;,\;\right\}}_{\mathrm{gc}}$ noncanonical. Evidently, μ is a Casimir invariant. The level set of μ, a leaf of the Casimir invariant, identifies the *macro hierarchy*. By applying Liouville’s theorem to the Poisson bracket ${\left\{\;,\;\right\}}_{\mathrm{gc}}$, the invariant measure on the macro hierarchy is $d^{4}z=d^{6}z/\left(2\pi d\mu \right)$.

This example suggests a physical origin of what we call a Casimir invariant. Unlike usual invariants in Hamiltonian mechanics, which are connected to some “symmetry” of a specific Hamiltonian, a Casimir invariant does not depend on the choice of Hamiltonian, so it is independent of any particular symmetry of matter. Instead, the invariance of a Casimir is due to the algebraic structure of the Poisson manifold. It is connected to some symmetry of the space, appearing as a nullity of the Poisson operator; cf. Remark 1. In that, adiabatic invariants have the same property; their origin is in the *scale separation* (or coarse-graining) of some degree of freedom of the space, not in any specific symmetry of matter. From these observations, we propose an interpretation: *a Casimir invariant is an adiabatic invariant* [14,15].

#### 2.2.3. Thermal Equilibrium on Macro Hierarchy

The most probable state on the macroscopic ensemble maximizes the entropy evaluated by the corresponding invariant measure. The variational principle is set up with the help of Lagrange multipliers; we maximize the entropy $S=-\int f\log f\;d^{6}z$ for a given particle number $N=\int fd^{6}z$, a quasi-particle number $M=\int \mu fd^{6}z$, and an energy $E=\int H_{\mathrm{gc}}fd^{6}z$, to obtain the distribution function

(14) $$f=\frac{e^{-\beta H_{\mathrm{gc}}-\alpha \mu }}{Z},$$

where α, β, and logZ−1 are, respectively, the Lagrange multipliers on *M*, *E*, and *N*. In this *grand canonical distribution function*, α/β is a chemical potential associated with the quasi-particles, which are the *guiding centers* of magnetized particles.

The factor $e^{-\alpha \mu }$ in $f_{\alpha }$ yields a direct $\omega_{c}$ dependence of the configuration space density:

(15) $$\rho =\int f\;\frac{2\pi \omega_{c}}{m}d\mu dv_{d}dv_{\|}\propto \frac{\omega_{c}\left(x\right)}{\beta \omega_{c}\left(x\right)+\alpha }.$$

**Figure 1:**
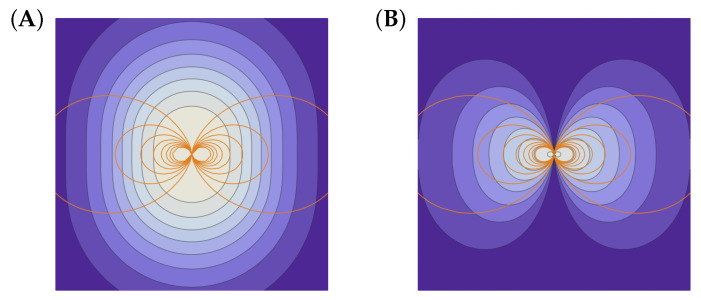
(**A**) The equilibrium on the leaf defined by μ. (**B**) The equilibrium on the leaf defined by μ and $J_{\|}$. Contours: Density distribution of magnetized plasma in the neighborhood of a point dipole. Curves: Magnetic field lines (level sets of ψ). See [14].

Notice that the Jacobian $(2\pi \omega_{c}/m)d\mu$ multiplying the macroscopic measure $d^{4}z$ reflects the distortion of the macro phase space (Casimir leaf) caused by the inhomogeneous magnetic field. Figure 1A shows the density distribution and the magnetic field lines.

The same argument applies when we further separate the bounce action–angle variables, assuming the constancy of $J_{\|}$ [14]; see Figure 1B.

The scale hierarchy is selected by the space-time scale of driving fluctuations; an adiabatic invariance holds when the corresponding frequency is higher than the frequency range of the fluctuations.

## 3. Hamiltonian Structure of Magnetofluid System

### 3.1. Hall-MHD System

Some variety of magnetofluid (plasma) systems, at the ideal (dissipation-free) limit, are known to be noncanonical Hamiltonian. Here, we invoke the Hall MHD system (to be called the H-MHD system), which is more adequate (indeed, simple, as will be shown later) to describe the dynamics of the magnetic field; in the usual MHD system, which omits the Hall term in the magnetic field equation, which is a particular singular limit of the H-MHD system, magnetic field and fluid elements are constrained to move together, causing the somewhat complex coupling of basic state variables.

Here we reset notation of some variables in order to avoid a lack of symbols. The naïve form of the governing equations is, denoting by ρ the mass density, by *s* the specific entropy (entropy in a volume element divided by ρ), by *p* the pressure, by V the fluid velocity, and by B the magnetic field,

(16) $$\begin{array}{ccc} & & \partial_{t}\rho +\nabla \cdot \left(V\rho \right)=0, \end{array}$$

(17) $$\begin{array}{ccc} & & \partial_{t}s+V\cdot \nabla s=0, \end{array}$$

(18) $$\begin{array}{ccc} & & \partial_{t}V-V\times \left(\nabla \times V\right)+\rho^{-1}\left(\nabla \times B\right)\times B=-\nabla \left(V^{2}/2\right)-\rho^{-1}\nabla p, \end{array}$$

(19) $$\begin{array}{ccc} & & \partial_{t}B-\nabla \times \left[\left(V-\epsilon \rho^{-1}\nabla \times B\right)\times B\right]=0. \end{array}$$

The variables are normalized in standard Alfvén units, i.e., energy densities (thermal E, kinetic $\rho V^{2}/2$ and magnetic $B^{2}/2\mu_{0}$) are normalized by the representative magnetic energy density $B_{0}^{2}/\mu_{0}$. We assume a thermodynamic relation $dh=Tds+\rho^{-1}dp$ with a specific thermal energy E(ρ,s), a temperature T=∂E/∂s, and specific enthalpy h=∂(ρE)/∂ρ. The *Hall parameter* $\epsilon =(c/\omega_{pi})/L$ ($c/\omega_{pi}$ is the ion skin depth, and *L* is the system size) scales the Hall term $\epsilon \rho^{-1}\left(\nabla \times B\right)\times B$ which works as a singular perturbation; in the limit of ϵ→0, the H-MHD system reduces to the MHD system.

To avoid complexity due to boundary conditions, we consider a periodic domain $M=T^{3}$, on which the Hamiltonian (energy) has a finite value.

We may put the H-MHD system (16)–(19) into a Hamiltonian form [12]. The state vector is $u{=}^{\;t}\left(\rho ,s,V,B\right)$, which is a member of the phase space $V=L^{2}\left(M\right)$ (so 〈,〉 is the $L^{2}$ inner product). We define

(20) $$\begin{array}{ccc} H_{\mathrm{mf}} & = & \int_{M}\left\{\left[\frac{V^{2}}{2}+E\left(\rho ,s\right)\right]\rho +\frac{B^{2}}{2}\right\}\;d^{3}x, \end{array}$$

(21) $$\begin{array}{ccc} J_{\mathrm{mf}} & = & \left(\begin{array}{cccc} 0 & 0 & -\nabla \cdot & 0\\ 0 & 0 & -\rho^{-1}\left(\nabla s\right)\cdot & 0\\ -\nabla & \rho^{-1}\nabla s & -\rho^{-1}\left(\nabla \times V\right)\times & \rho^{-1}\left(\nabla \times \circ \right)\times B\\ 0 & 0 & \nabla \times \left(\circ \times \rho^{-1}B\right) & -\epsilon \nabla \times [\rho^{-1}\left(\nabla \times \circ \right)\times B] \end{array}\right). \end{array}$$

Inserting $\partial_{u}H_{\mathrm{mf}}={\left(V^{2}/2+h,\rho T,\rho V,B\right)}^{T}$, we observe that the corresponding Hamilton’s equation

(22) $$\dot{u}=J_{\mathrm{mf}}\left(u\right)\partial_{u}H_{\mathrm{mf}}\left(u\right)$$

reproduces the H-MHD Equations (16)–(19). However, the Poisson operator (21) is considerably complex, and indeed it is noncanonical.

We find three independent Casimir invariants:

(23) $$\begin{array}{ccc} C_{0} & = & \int_{M}\rho \;d^{3}x, \end{array}$$

(24) $$\begin{array}{ccc} C_{1} & = & \int_{M}s\rho \;d^{3}x, \end{array}$$

(25) $$\begin{array}{ccc} C_{2} & = & \frac{1}{2}\int_{M}A\cdot B\;d^{3}x, \end{array}$$

where A is the vector potential ($A={\mathrm{curl}}^{-1}B$, with a compact self-adjoint operator ${\mathrm{curl}}^{-1}$ [27]). These Casimir invariants have distinct physical meaning; $C_{0}$ is the total mass, $C_{1}$ is the total entropy, and $C_{2}$ is the magnetic helicity.

**Remark** **2** (Barotropic flow and fluid helicity). *When E=E(ρ) and independent of s, the right-hand side of (18) reduces into an exact differential $-\nabla (h+V^{2}/2)$. Then, the baroclinic term $-\nabla \rho^{-1}\times \nabla p=\nabla T\times \nabla s$ (due to the non-exact differential T∇s) vanishes in the vorticity equation, that is, the curl of (18); such a fluid (or a plasma) is said to be barotropic. The baroclinic term represents the thermodynamic mechanism of generating vorticity; in its absence, another Casimir invariant (called canonical fluid helicity) emerges:*

(26) $$C_{3}=\frac{1}{2}\int_{M}P\cdot \left(\nabla \times P\right)\;d^{3}x,$$

*where*

(27) $$P=V+\epsilon^{-1}A,$$

*is a “canonical momentum” with an effective charge $\epsilon^{-1}$. However, the baroclinic term violates the constancy of the canonical fluid helicity $C_{3}$:*

(28) $${\dot{C}}_{3}=\int_{M}\left(T\nabla s\right)\cdot \left(\nabla \times P\right)\;d^{3}x.$$

*Simply by putting B=0 in the H-MHD system (16)–(19), we obtain a neutral fluid model, which has Casimir invariants $C_{0}$ and $C_{1}$, but $C_{2}$ trivializes. When the barotropic relation holds, the fluid helicity $C_{3}^{\prime }=\frac{1}{2}\int_{M}V\cdot \left(\nabla \times V\right)\;d^{3}x$ conserves.*

*With the canonical vorticity ω=∇×P, Equation (26) reads*

$$C_{3}=\frac{1}{2}\int_{M}\left({\mathrm{curl}}^{-1}\omega \right)\cdot \omega \;d^{3}x.$$

*which parallels the magnetic helicity (25) by the correspondence of B and ω. The essential element common to these “helicities” is the integral operator ${\mathrm{curl}}^{-1}$, which includes a singularity $1/|x-x^{\prime }|$. Recall that this singularity is the origin of the “residues” in complex (two-dimensional) integrals, which is extended to three-dimensional integrals as Gauss’ linking numbers. The reader is referred to [28,29,30,31,32] for the “topological” meanings of helicity and its role in fluid/plasma theory.*

### 3.2. Canonical Representation of H-MHD System

The conservation of the Hamiltonian (energy) $H_{\mathrm{mf}}$ is due to the temporal symmetry $\partial_{t}H_{\mathrm{mf}}=0$. In contrast, the conservation of a Casimir invariant is due to the Poisson operator, being independent of the choice of Hamiltonian, i.e., Casimir invariants are not related to any explicit symmetry of matter (cf. Remark 1). A possible mechanism that yields a Casimir invariant is a *gauge symmetry* in some representation of V, and then a Casimir invariant is a Noether charge of the gauge symmetry.

Suppose that there is an “underlying” phase space Z of fundamental variables ζ, with the physical variables u represented by some specific combinations of ζ, where the map ζ↦u is redundant. Then, a gauge freedom occurs. Here, the relevant symmetry is that of the *Clebsch parameterization* [13,16,17,18,19,20,21,22,23] for which it was shown that the helicities are Noether charges [33].

We invoke this picture in order to formulate an appropriate *invariant measure* on a canonical phase space, and view the H-MHD system as constrained (reduced) mechanics on a foliated symplectic manifold (which we call a *Casimir leaf*). The following formulation is a slight generalization of the previous model [12] in order to incorporate the baroclinic effect.

We consider a 12-component vector field

(29) $$\zeta ={\left(\varphi ,\varrho ,\sigma ,\mu ,q^{1},p_{1},q^{2},p_{2},\theta^{1},\psi_{1},\theta^{2},\psi_{2}\right)}^{T}\;\in Z,$$

where the odd-number components ($\varphi ,\sigma ,q^{j},\theta^{j}$; j=1,2) are 0-forms (scalars), while the even-number components ($\varrho ,\mu ,p_{j},\psi_{j}$; j=1,2) are 3-forms in the base space $M=T^{3}$. We assume $\zeta_{n}$ (n=1,⋯,12) are smooth (i.e., $C^{\infty }$-class) functions. The dual space $Z^{*}$ is the Hodge dual of Z, i.e., the odd number components of $\eta \in Z^{*}$ are *n*-forms and the even number components are 0-forms. The pairing of $Z^{*}$ and Z is

(30) $$\langle \eta ,\zeta \rangle =\sum_{n}\int_{M}\eta^{n}\wedge \zeta_{n},\;\eta \in Z^{*},\;\zeta \in Z.$$

On the space $C^{\infty }\left(Z\right)$ of *observables*, we define a canonical Poisson bracket

(31) $$\left\{F,G\right\}=\langle \partial_{\zeta }F,J_{c}\partial_{\zeta }G\rangle ,$$

where $F,G\in C^{\infty }\left(Z\right)$, $\partial_{\zeta }F\;\left(\in Z^{*}\right)$ is the gradient of *F*, and $J_{c}:\;Z^{*}\to Z$ is the symplectic operator (with $J_{c}$ of (5)):

(32) $$J_{c}=J_{c}⊕J_{c}⊕J_{c}⊕J_{c}⊕J_{c}⊕J_{c}.$$

The adjoint representation of the Poisson algebra gives the determining Equation (3) of the dynamics.

We relate the physical quantity u∈V and ζ∈Z as follows. We identify

(33) $$\left\{\begin{array}{ccc} \rho & \Leftrightarrow & \varrho \\ s & \Leftrightarrow & \sigma \\ V & \Leftrightarrow & -\varrho^{-1}\left(\varrho d\varphi +\mu d\sigma +p_{j}dq^{j}+\psi_{j}d\theta^{j}\right)\\ A & \Leftrightarrow & A=\epsilon \varrho^{-1}\psi_{j}d\theta^{j}, \end{array}\right.$$

where index *j* runs over {1,2}, and we apply the summation rule. To simplify notation, we define scalars

$$\overset{ˇ}{\mu }=\frac{\mu }{\varrho },\;\overset{ˇ}{p_{j}}=\frac{p_{j}}{\varrho },\;\overset{ˇ}{\psi_{j}}=\frac{\psi_{j}}{\varrho }\;\left(j=1,2\right).$$

A rather complicated expression of the fluid (ion) velocity V comes from the effective momentum

$$℘=V+\epsilon^{-1}A+\overset{ˇ}{\mu }\nabla \sigma \;\Leftrightarrow \;-\left(d\varphi +\overset{ˇ}{p_{j}}dq^{j}\right).$$

In the second expression, the first two terms are the canonical momentum of (27), and the last term is pertinent to a thermal force. The magnetic field is parameterized as $B=\epsilon \nabla \overset{ˇ}{\psi_{j}}\times \nabla \theta^{j}$. Writing the physical quantities u as (33) is called the *Clebsch parameterization* [11,16]. It is known that five Clebsch parameters $\varphi ,{\overset{ˇ}{p}}_{j},q^{j}$ suffice for representing every 3-vector *℘*, and four Clebsch parameters ${\overset{ˇ}{\psi }}_{j},\theta^{j}$ for every divergence-free 3-vector B [22].

Inserting (33) into the energy $H_{\mathrm{mf}}$ of (20), we obtain the Hamiltonian represented by the Clebsch parameters (hereafter, we use vector analysis notation, but the corresponding meaning in differential geometry may be clear):

(34) $$\begin{array}{ccc} H_{c}\left(\zeta \right) & = & \int_{M}\left\{\left[\frac{{\left|\nabla \varphi +\overset{ˇ}{\mu }d\sigma +{\overset{ˇ}{p}}_{j}\nabla q^{j}+{\overset{ˇ}{\psi }}_{j}\nabla \theta^{j})\right|}^{2}}{2}+E\left(\varrho ,\sigma \right)\right]\varrho \right.\\ & & \;\;\;\;\;\;\;\;\;\;\;\;\;\;\;\;\;\;\;\;\;\;\;\;\;\;\;\;\;\;\;\;\;\;\;\;\;\;\;\;\;\;\;\;\;\left.+\frac{{\left|\epsilon \nabla {\overset{ˇ}{\psi }}_{j}\times \nabla \theta^{j}\right|}^{2}}{2}\right\}\;d^{3}x. \end{array}$$

With this $H_{c}$, the canonical Hamilton’s equation $\dot{\zeta }=J_{c}\partial_{\zeta }H_{c}$ reads

(35) $$\begin{array}{ccc} \dot{\varphi } & = & \partial_{\varrho }H_{c}=-\left(V\cdot \nabla \right)\varphi +h-V^{2}/2-\rho^{-1}J\cdot A, \end{array}$$

(36) $$\begin{array}{ccc} \dot{\rho } & = & -\partial_{\varphi }H_{c}=-\nabla \cdot \left(V\varrho \right), \end{array}$$

(37) $$\begin{array}{ccc} \dot{\sigma } & = & \partial_{\mu }H_{c}=-\left(V\cdot \nabla \right)\sigma , \end{array}$$

(38) $$\begin{array}{ccc} \dot{\mu } & = & -\partial_{\sigma }H_{c}=-\nabla \cdot \left(V\mu \right)-\rho T, \end{array}$$

(39) $$\begin{array}{ccc} {\dot{q}}^{j} & = & \partial_{p_{j}}H_{c}=-\left(V\cdot \nabla \right)q_{j}, \end{array}$$

(40) $$\begin{array}{ccc} {\dot{p}}_{j} & = & -\partial_{q^{j}}H_{c}=-\nabla \cdot \left(Vp_{j}\right), \end{array}$$

(41) $$\begin{array}{ccc} {\dot{\theta }}^{j} & = & \partial_{\psi_{j}}H=-\left(V_{e}\cdot \nabla \right)\theta^{j}, \end{array}$$

(42) $$\begin{array}{ccc} {\dot{\psi }}_{j} & = & -\partial_{\theta^{j}}H=-\nabla \cdot \left(V_{e}\psi_{j}\right), \end{array}$$

where $V_{e}$ is the electron velocity defined by (with the total current density J=∇×B)

(43) $$V_{e}=V-\epsilon \rho^{-1}J.$$

Evidently, Equations (36) and (37) are, respectively, the mass conservation law (16) and the entropy conservation law (17). The rest of the H-MHD equations, Equations (18) and (19), are reproduced by evaluating $\partial_{t}V$ and $\partial_{t}\left(\nabla \times A\right)$ in terms of the Clebsch parameters (33) with (35)–(41). Hence, the canonical Hamilton’s equation with the Clebsch-parameterized Hamiltonian (34) describes the plasma dynamics obeying H-MHD equations [12].

It should be added that the canonicalization by Clebsch parameterization also provides a natural path to the quantization of a Poisson manifold; cf. [13,21].

### 3.3. Dynamics Observed in Base-Space

The canonicalized H-MHD system (35)–(42) elucidates deep geometric meanings of plasma dynamics. Let $L_{v}$ be the conventional Lie derivative along the vector v∈TM, i.e., by Cartan’s formula,

(44) $$L_{v}=di_{v}+i_{v}d.$$

Interestingly, the H-MHD system (35)–(42) is characterized by two different vectors V (ion flow) and $V_{e}$ (electron flow). Using the Lie derivatives, we may rewrite the canonical equations as

(45) $$\begin{array}{cc} \left\{\begin{array}{c} \left(\partial_{t}+L_{V}\right)\varphi =h-V^{2}/2-\rho^{-1}J\cdot A,\\ (\partial_{t}+L_{V})\sigma =0,\\ \left(\partial_{t}+L_{V}\right)q^{j}=0,\\ \left(\partial_{t}+L_{V_{e}}\right)\theta^{j}=0, \end{array}\right.\; & \;\;\;\; \end{array}\left\{\begin{array}{c} (\partial_{t}+L_{V})\varrho =0,\\ (\partial_{t}+L_{V})\mu =-\rho T,\\ \left(\partial_{t}+L_{V}\right)p_{j}=0,\\ \left(\partial_{t}+L_{V_{e}}\right)\psi_{j}=0. \end{array}\right.$$

Remember that $\zeta_{2n-1}$, summarized in the left group, are scalars, while $\zeta_{2n}$, in the right group, are 3-forms. We observe that all Clebsch variables, excepting φ and μ, are just *Lie-dragged* (conserved along the flow as scalars or 3-forms); the ion-related variables ($\varrho ,\sigma ,q^{j},p_{j}$) are moved by the ion flow velocity V, while the magnetic-field variables ($\theta^{j},\psi_{j}$) are moved by the electron flow velocity $V_{e}$.

**Remark** **3** (Dual vectors and current). To be precise, V and $V_{e}$ in the Lie derivatives of (45) are “vectors” $V^{†}$ and $V_{e}^{†}\in TM$, which are the deal of the covectors (1-forms $\in T^{*}M$) defined in (33). We interpret (43) as a 2-form relation $i_{V_{e}^{†}}\varrho =i_{V^{†}}\varrho -\epsilon *J$, where J=δdA and its Hodge dual *J is the conventional current density J (2-form). In (35), $V^{2}=i_{V^{†}}V$, and J·A=*J∧A, which is divided by ϱ to yield a scalar (*J∧A)/ϱ.

These relations reveal a more local mechanism behind the conservation of the Casimir invariants, by which we can develop a “particle model”. Let $\Omega_{i}\left(t\right)$ and $\Omega_{e}\left(t\right)$ be three-dimensional subdomains (⊂M) moved, respectively, by the vector V and $V_{e}$. The integral over $\Omega_{i}\left(t\right)$ of the Lie-dragged 3-form ϱ, i.e.,

(46) $$C_{0}\left(\Omega_{i}\right)=\int_{\Omega_{i}\left(t\right)}\varrho =\int_{\Omega_{i}\left(t\right)}\rho \;d^{3}x,$$

is a constant of motion, which is the mass contained in the co-moving subdomain $\Omega_{i}\left(t\right)$. The Casimir invariant $C_{0}$ is the special case: $C_{0}=C_{0}\left(M\right)$.

There are two other 3-forms dragged by the electron velocity:

(47) $$\begin{array}{ccc} C_{1}\left(\Omega_{e}\right) & = & \frac{1}{2}\int_{\Omega_{e}\left(t\right)}\sigma \varrho =\frac{1}{2}\int_{\Omega_{e}\left(t\right)}s\rho \;d^{3}x, \end{array}$$

(48) $$\begin{array}{ccc} C_{2}\left(\Omega_{e}\right) & = & \frac{1}{2}\int_{\Omega_{e}\left(t\right)}A\wedge dA=\frac{1}{2}\int_{\Omega_{e}\left(t\right)}A\cdot B\;d^{3}x, \end{array}$$

which are the “local” entropy and magnetic helicity. When we take $\Omega_{e}\left(t\right)=M$, we obtain $C_{1}=C_{1}\left(M\right)$ and $C_{2}=C_{2}\left(M\right)$.

These constants of motion can be regarded as *topological charges* (we say “topological” because they are invariant under the group action generated by the flow) characterizing the *particles* $\Omega_{i}\left(t\right)$ and $\Omega_{e}\left(t\right)$. Both particles move with different velocities V and $V_{e}$, respectively.

## 4. Kinetic Theory with Topological Constraints

### 4.1. Co-Adjoint Representation of the Canonicalized H-MHD Dynamics

By the canonical Poisson operator $J_{c}$, the adjoint representation of the Poisson algebra ${\mathrm{ad}}_{H_{c}}\circ =\left\{\circ ,H_{c}\right\}=\langle \partial_{\zeta }\circ ,J_{c}\partial_{\zeta }H_{c}\rangle$ dictates the dynamics of the observables in $g=C_{\left\{\;,\;\right\}}^{\infty }\left(Z\right)$. In this section, we formulate the corresponding *co-adjoint representation* by introducing a *distribution function* on the phase space Z. A distribution function measures the statistical average of observables (∈g), so it lives in the dual space $g^{*}$. We are going to construct a Poisson manifold $G=C^{\infty }\left(g^{*}\right)$ in order to describe the dynamics of distribution functions. Such a Poisson algebra was first studied by Lie in the 19th century [34,35,36,37,38,39], which was indeed the origin of Poisson algebras. The idea was to consider the co-adjoint representation of a given Lie algebra. Here, the H-MHD canonical Poisson algebra $g=C_{\left\{\;,\;\right\}}^{\infty }\left(Z\right)$ is the starting point, so $G=C^{\infty }\left(g^{*}\right)$ may be regarded as a *hyper Poisson algebra* over the canonical H-MHD system.

Let 〈〈,〉〉 denote a pairing of $g\times g^{*}\to R$ (the conjugate elements $\in g^{*}$ go to the right-hand side), which must be distinguished from the previous $\langle \;,\;\rangle :\;Z^{*}\times Z\to R$. The concrete form of 〈〈,〉〉 (with an explicit definition of the dual space $g^{*}$) will be given in the next subsection; here we proceed with an abstract definition. We denote by *f* an element of $g^{*}$. For $G\left(f\right)\in C^{\infty }\left(g^{*}\right)$, we define its *gradient* $\partial_{f}G\in g$ by

(49) $$\delta G=G\left(f+\epsilon \tilde{f}\right)-G\left(f\right)=\epsilon \langle \;\langle \;\partial_{f}G,\tilde{f}\;\rangle \;\rangle +O\left(\epsilon^{2}\right)\;\left(\forall \tilde{f}\in g^{*}\right).$$

Regarding $f\left(\zeta \right)\in g^{*}$ as a *distribution function* (representing a mixed state of H-MHD state vectors ζ∈Z; see the next subsection), we construct a “hyper Poisson manifold” $C^{\infty }\left(g^{*}\right)$ by endowing it with

(50) $$\{\;\{\;G,H\;\}\;\}=\langle \;\langle \;\left\{\partial_{f}G,\partial_{f}H\right\},f\;\rangle \;\rangle =\langle \;\langle \;\partial_{f}G,{\left\{\partial_{f}H,f\right\}}^{*}\;\rangle \;\rangle ,$$

where ${\left\{\;,\;\right\}}^{*}:\;g\times g^{*}\to g^{*}$ is the dual representation of {,}. Because of this construction, {{,}} inherits bilinearity, anti-symmetry, and the Jacobi’s identity from that of {,}. The Leibniz property is explicitly implemented by the derivation $\partial_{f}$, so {{,}} defines a Poisson algebra on $C^{\infty }\left(g^{*}\right)$. However, we note that the formal bracket ${\left\{\;,\;\right\}}^{*}$ is not necessarily a Lie bracket.

The dynamics in the hyper Poisson manifold $C^{\infty }\left(g^{*}\right)$ dictates the evolution of the distribution function f(ζ):

(51) $$\dot{G}=\{\;\{\;G,H\;\}\;\},$$

which reads, by $\dot{G}=\langle \;\langle \;\partial_{f}G,\dot{f}\;\rangle \;\rangle$,

(52) $$\dot{f}={\left\{\partial_{f}H,f\right\}}^{*}.$$

The action of ${\mathrm{ad}}_{H}^{*}\circ ={\left\{\partial_{f}H,\circ \right\}}^{*}$ on the distribution function *f* may be viewed as the co-adjoint representation of the Poisson algebra g.

### 4.2. Distribution Function

Here, we provide the forgoing abstract formulation with a concrete form, and make ${\mathrm{ad}}_{H}^{*}$ explicit. We have to define the dual space $g^{*}$ and the pairing 〈〈,〉〉 explicitly.

An element $G\left(\zeta \right)\in g=C^{\infty }\left(Z\right)$ is regarded as a functional of ζ that is a smooth section of the vector bundle over the base space *M*; each fiber ($\pi^{-1}\left(x\right)$ for x∈M; π is the projection) is a 12-dimension symplectic vector space, whose coordinate will be denoted by $\eta ={\left(\eta_{1},\eta_{2},\cdots ,\eta_{11},\eta_{12}\right)}^{T}$. We may write

(53) $$G\left(\zeta \right)=\int_{M}{g\left(\zeta \right)|}_{x}\;d^{3}x$$

with a real-valued function g(ζ); see the Hamiltonian (34) as an explicit example. We note that ${g\left(\zeta \right)|}_{x}$ (denoting the value of g(ζ) at the position x∈M) is not necessarily determined only by the local value ζ(x), but it may depend on the whole structure of the section ζ (as the derivatives of ζ are included in g(ζ)). We may rewrite (53) as

(54) $$\begin{array}{ccc} G(\zeta ) & = & \int_{M}\int_{Z}{g\left(\eta \right)|}_{x}\delta \left(\eta -\zeta \right)\;d\eta \;d^{3}x,\\ & = & \int_{M}\int_{M}\int_{\pi^{-1}\left(y\right)}{g\left(\eta \left(y\right)\right)|}_{x}\delta \left(\eta \left(y\right)-\zeta \left(y\right)\right)\;d\eta \left(y\right)d^{3}y\;d^{3}x, \end{array}$$

which evaluates G(ζ) for a specific section η=ζ of the vector bundle. In the detailed expression, given the second line of (54), η(y) denotes the coordinate on the fiber $\pi^{-1}\left(y\right)$ (y∈M).

We can generalize (54) for a *mixed state* that is a bunch of sections $\zeta_{1},\cdots ,\zeta_{N}$ (*N* is a certain integer). With

(55) $$f\left(\eta \right)=\int_{M}f_{x}\left(\eta \right)\;d^{3}x,\;f_{x}\left(\eta \right)=\frac{1}{N}\sum_{\ell =1}^{N}\delta \left(\eta -\zeta_{\ell }\left(x\right)\right),$$

we define

(56) $$G\left(f\right)=\langle \;\langle \;G,f\;\rangle \;\rangle =\frac{1}{N}\sum_{\ell =1}^{N}\int_{M}g\left(\zeta_{\ell }\right){|}_{x}\;d^{3}x,$$

Now the meaning of $g^{*}$ is clear; it is the space of *distributions* specifying the sections of the vector bundle Z constituting a mixed state. By (49), we observe $\partial_{f}G=G$.

For G(f) and H(f) ($\in G=C^{\infty }\left(g^{*}\right)$), we write $\partial_{f}G\left(f\right)=G\left(\zeta \right)$ and $\partial_{f}H\left(f\right)=H\left(\zeta \right)$ ($\in g=C^{\infty }\left(Z\right)$). Then, we observe

(57) $$\begin{array}{ccc} \left\{\;\{\;G,H\;\}\;\right\} & = & \langle \;\langle \;\{G,H\},f\;\rangle \;\rangle \\ & = & \int_{M}\int_{Z}\left(\partial_{\eta }G\cdot J_{c}\partial_{\eta }H\right){|}_{x}\;f\left(\eta \right)\;d\eta \;d^{3}x\\ & = & \int_{M}\int_{Z}g\left(\eta ,x\right)\;\left(\partial_{\eta }H\cdot J_{c}\partial_{\eta }f\right){|}_{x}\;d\eta \;d^{3}x=\langle \;\langle \;G,{\left\{H,f\right\}}^{*}\;\rangle \;\rangle . \end{array}$$

Here we use the identity $\partial_{\eta }\left[J_{c}\partial_{\eta }H\left(\eta \right)\right]=0$ (∀H(η)∈g), and the compactness of f(η) yielding $\int_{Z}\partial_{\eta }\left[F\left(\eta \right)f\left(\eta \right)\right]\;d\eta$ (∀F(η)∈g). Hence, ${\left\{\;,\;\right\}}^{*}$ is formally equivalent to {,} (as known in conventional kinetic theory). The co-adjoint representation (52) of H-MHD dynamics is now written as a field-theoretic (infinite-dimensional) kinetic equation; with the Clebsch parameterized Hamiltonian $H_{c}$ of (34)

(58) $$\dot{f}={\left\{H_{c},f\right\}}^{*}.$$

**Remark** **4** (Particle model). *In analogy to the Vlasov equation of the plasma kinetic theory (cf. [34]), the distribution function f may be regarded as a statistical distribution of “particles” representing some localized elements in the phase space. The conventional idea of such particles are the Fourier modes living in the function space (cf. Remark 5). However, we can proffer alternatives by our framework. A smart definition of particles $\left\{\zeta_{\ell };\;\ell =1,\cdots ,N\right\}$ is given by the particle picture described in Section 3.3, which discretizes the base space M, instead of the wave-number space. Let $\Omega_{\ell }$ (ℓ=1,⋯,N) be a set of disjoint subdomains covering M, and $I_{\ell }$ be the indicator, i.e.,*

$$I_{\ell }=\left\{\begin{array}{cc} 1 & \mathrm{if}\;x\in \Omega_{\ell },\\ 0 & \mathrm{if}\;x\notin \Omega_{\ell }. \end{array}\right.$$

*We set the initial condition as*

(59) $$\zeta_{\ell }=I_{\ell }\zeta \;\left(\ell =1,\cdots ,N\right).$$

*At t>0, each $\Omega_{\ell }$ branches into two different subdomains; the ion flow velocity V gives $\Omega_{\ell ,i}\left(t\right)$, while the electron flow velocity $V_{e}$ gives $\Omega_{\ell ,e}\left(t\right)$. As shown in Section 3.3, ρ, σ, $q^{j}$, and $p_{j}$ move with $\Omega_{\ell ,i}\left(t\right)$ so that the local mass $C_{0}$ and entropy $C_{1}$ stick to the ion flow. On the other hand, $\theta^{j}$ and $\psi_{j}$ move with $\Omega_{\ell ,e}\left(t\right)$ so that the local magnetic helicity $C_{2}$ stick to the electron flow. The complexity of dynamics (often called turbulence) arises from the complicated involvement of all Clebsch parameters in the determination of V and $V_{e}$.*

**Remark** **5** (Comparison with other statistical models of turbulence). *Here we compare our statistical theory with other typical models of fluid/plasma turbulences (for an intensive review of plasma turbulence, see [40]). The conventional idea for defining a statistical ensemble is the “Fourier decomposition” of field variables V, B, etc. (in contrast, our ensemble $\left\{\zeta_{\ell }\right\}$ is typically a “spatial decomposition” into what we call particles; see (59) and Remark 4). For a rectangular periodic domain, we can apply the standard Fourier decomposition of field variables to span the phase space. For a general domain, which may possibly be multiply connected, a sensible idea is to invoke the Beltrami fields [41,42], i.e., the eigenfunctions of the curl operator [27]. The nonlinearity of the evolution equation brings about mode coupling among the decomposed components.*

*There are two categories of approaches to analyzing statistical equilibria. The so-called “cascade model” assumes an open system in which kinetic energy is injected on a large scale and dissipated (removed) on a small scale, and estimates the energy transfer rate in the mode coupling to derive the spectrum (distribution of energy among modes of different scales). After the pioneering work of Kolmogorov [43,44], a variety of models have been studied for different systems; for example, see review paper [45] for two-dimensional systems.*

*The other category consists of statistical mechanical models of various ensembles. When considering the distribution function f, which is a probability “density” in some phase space Z, one has to provide Z with a “measure” μ by which the integral $\int_{E}fd\mu$ over an arbitrary domain E⊂Z evaluates the probability of a state staying in E. For the conservation of probability, such a measure must be invariant over all possible transitions, i.e., the volume $\int_{E}d\mu$ of every E⊂Z must be conserved when E moves together with the transition of states. This means that Liouville’s theorem must apply to the underlying dynamics law (as we have demonstrated for our canonicalized Hamiltonian system in Section 3.2). In the model of incompressible fluids, we can show $\partial {\dot{c}}_{\ell }/\partial c_{\ell }=0$ for every mode amplitude $c_{\ell }$, which implies that the transition in the phase space is incompressible so that Liouville’s theorem applies (for example, see [41,45]). A subtle problem is the “ultraviolet catastrophe” (known for the blackbody radiation) caused by the infinite dimension of the phase space. A possible recourse is the second quantization similar to quantum field theory [41]. Another easy method is to truncate the phase space into a finite-dimension space, which of course violates the exact Liouville’s theorem, but lower-order invariants, such as energy and helicity, can be maintained (cf. [40,46,47]). In contrast, our formulation is free from such arguments; the statistical ensemble is a set of purely virtual copies of the system, represented by co-adjoint orbits, and thus reflects the precise Poisson structure.*

### 4.3. Boltzmann Distribution

We are ready to describe the statistical mechanics of the H-MHD system in the canonicalized phase space Z. While the kinetic Equation (58) is temporally reversible, a statistical model needs an appropriate dissipation term (or a “collision term”) that describes irreversible entropy production. Because the aim of this work is to characterize the equilibrium state, we do not go further into the explicit formulation of an appropriate dissipation term but only delineate the fundamental requirements.

The issue is how the invariance of Casimirs (or Noether charges in the canonicalized formulation) can be maintained while the dissipation admits the violation of microscopic topological constraints. As discussed in Section 3.3, most of the Clebsch parameters are Lie-dragged in the base space; hence, there are many topological constraints pertaining to the local points, curves, surfaces, and volumes, moved by either V or $V_{e}$ ($C_{0}$, $C_{1}$ and $C_{2}$ are such quantities pertaining to volume elements and the corresponding 3-forms; cf. Remark 4). A dissipation mechanism can break such topological constraints that prevent the *rearrangement* of physical quantities, leading to the statistical equilibrium. On the other hand, however, the total values of mass, helicity, and energy must be conserved (we start with a microcanonical ensemble), and hence, appropriate constraints must be imposed to restrict the range of the transition probability. This leads to the idea of an *energy–Casimir shell*, which is a generalization of the conventional energy shell, i.e., we consider the level set of the total energy and total Casimir invariants (excepting the thermal entropy $C_{1}$) in the phase space. Since the energy $H_{c}\left(\eta \right)$ and the helicity $C_{2}\left(\eta \right)$ are rather complex functionals of η, it is not easy to write the collision term on the energy–Casimir shell. However, the method of the Lagrange multiplier helps to calculate the maximum entropy on the energy–Casimir shell.

Maximizing the entropy, which measures the probability of the distribution over the Poisson manifold $g=C_{\left\{\;,\;\right\}}^{\infty }\left(Z\right)$ (notice the difference from the thermal entropy *s*),

(60) $$S=-\int_{M}\int_{Z}f_{x}\left(\eta \right)\log f_{x}\left(\eta \right)\;d\eta d^{3}x$$

for a fixed energy $E=\langle \;\langle \;H_{c},f\;\rangle \;\rangle$, helicity $W=\langle \;\langle \;C_{2},f\;\rangle \;\rangle$, and the total probability 1=〈〈1,f〉〉, we obtain the Boltzmann distribution

(61) $$f_{x}=\frac{1}{Z}e^{-\beta (h_{c}+\lambda c_{2})},$$

where $h_{c}$ and $c_{2}$ are, respectively, the integrand of $H_{c}$ and $C_{2}$. The normalizing factor *Z* is the partition function, β is the reciprocal of the *temperature* (which measures the randomness of the distribution *f*; different from the thermal temperature pertinent to the internal energy E), and λ is the *chemical potential* pertinent to the helicity; from the perspective of grand canonical ensemble, we may regard $C_{2}\left(\Omega_{e}\right)$ as a particle carrying helicity (see Section 3.3), and $C_{2}$ is the total number of such particles contained in *M*, and then, $\lambda c_{2}$ evaluates the energy brought about by the helicity particles.

At “low temperature” (β→∞), the most probable state condensates into the minimizer of $H_{c}+\lambda C_{2}$, which is the Taylor relaxed state [1,2].

## 5. Summary

General (noncanonical) Hamiltonian mechanics has a wide enough mathematical structure for describing “topological constraints” that plays an important role in the process by which non-trivial structures self-organize in opposition to the entropy principle. Here, such topological constraints appear as the non-trivial *center* of the governing Poisson algebra, i.e., non-zero element *C* such that {C,H}=0 for every *H* (notice that a canonical Hamiltonian system has only C=0 as the center). Such a *C*, called a Casimir invariant, is a constant of motion that cannot be changed by any energy *H*. When the phase space is foliated by a level set of a functional *C*, and a leaf has a non-trivial shape, the effective Hamiltonian, constrained on a leaf, may have a skewed distribution, giving rise to curious dynamics and structures.

In this paper, we have studied two examples; one (given in Section 2) is a finite dimensional system, and the other (given in Section 3 and Section 4) is an infinite dimensional system. In constructing a statistical mechanical theory, we need an appropriate metric to measure the probability density, i.e., we need Liouville’s theorem to guarantee the conservation of probabilities. In the first example, we have an explicit pullback relation for the coordinates on the leaf; see (15). However, in the second example, such a relation for infinite-dimensional phase spaces is not so simple. Yet, we can appeal to the method of the Lagrange multiplier to calculate the Boltzmann distribution under topological constraints represented by Casimir invariants.

The formulation of an appropriate dissipation (collision) term is still a challenging problem which is left for future study. Since the arguments on Jacobi’s identity, invariant measure, and the H-theorem have common roots in the symplectic geometry underlying canonical Hamiltonian systems, noncanonical systems (often encountered in macroscopic dynamics) require careful consideration when discussing entropy-generating processes; see, for example, [48]. As shown in Section 4, canonicalization by immersion in a symplectic manifold (cf. Remark 1) may provide a simpler description of the dynamics.

## Data Availability

The original contributions presented in this study are included in the article. Further inquiries can be directed to the corresponding author.
